# Oil painting teaching design based on the mobile platform in higher art education

**DOI:** 10.1038/s41598-024-65103-3

**Published:** 2024-07-05

**Authors:** Guodong Yi

**Affiliations:** Changsha Normal University, Changsha, 410151 Hunan China

**Keywords:** Deep learning, Artificial intelligence, Oil painting, Convolutional neural network, Support vector machine, Personalized teaching, Energy science and technology, Engineering, Materials science, Mathematics and computing, Nanoscience and technology, Optics and photonics, Physics

## Abstract

To improve the current oil painting teaching mode in Chinese universities, this study combines deep learning technology and artificial intelligence technology to explore oil painting teaching. Firstly, the research status of individualized education and related research on image classification based on brush features are analyzed. Secondly, based on a convolutional neural network, mathematical morphology, and support vector machine, the oil painting classification model is constructed, in which the extracted features include color and brush features. Moreover, based on artificial intelligence technology and individualized education theory, a personalized intelligent oil painting teaching framework is built. Finally, the performance of the intelligent oil painting classification model is evaluated, and the content of the personalized intelligent oil painting teaching framework is explained. The results show that the average classification accuracy of oil painting is 90.25% when only brush features are extracted. When only color features are extracted, the average classification accuracy is over 89%. When the two features are extracted, the average accuracy of the oil painting classification model reaches 94.03%. Iterative Dichotomiser3, decision tree C4.5, and support vector machines have an average classification accuracy of 82.24%, 83.57%, and 94.03%. The training speed of epochs data with size 50 is faster than that of epochs original data with size 100, but the accuracy is slightly decreased. The personalized oil painting teaching system helps students adjust their learning plans according to their conditions, avoid learning repetitive content, and ultimately improve students' learning efficiency. Compared with other studies, this study obtains a good oil painting classification model and a personalized oil painting education system that plays a positive role in oil painting teaching. This study has laid the foundation for the development of higher art education.

## Introduction

At present, the education mode of higher art education in China is mainly to inculcate students with knowledge^1^. Among them, the main forms of modern art education include offline one-to-one and one-to-many. Art teachers focus on the use of modern teaching methods and concepts to teach students the basic skills and knowledge of painting^2^. However, this teaching concept also has some shortcomings, and students' independent inquiry and learning abilities are easily ignored^3^. Oil painting teaching is a vital part of higher art education. Traditional oil painting teaching mode is not conducive to developing students' creativity and personality cultivation, and the teaching process lacks interactivity and interest^4^. The development of information technology (IT) provides new possibilities for oil painting teaching. Now, many researchers have combined artificial intelligence technology (AIT) with art teaching, involving applying artificial intelligence (AI) in the construction of learning resources, subject knowledge, and learning strategies, to help learners form individualized learning thinking, and develop personalized learning styles, take shape a unique art form for students^5^.

With the rapid progress of mobile network technology, mobile applications have penetrated people's lives. As the most commonly used intelligent mobile terminal, mobile phones play a vital role in daily life^6^. Traditional media can no longer meet users' demand for information according to a survey. A growing number of people use mobile terminals such as phones or iPads to obtain information^7^. For the past few years, intelligent teaching tools and applications based on mobile terminals have been developed and employed more and more widely, which has changed the schools’ teaching methods^8^. Mobile learning application reflects the application results of IT in mobile learning. If different links in the teaching process are properly utilized, it can improve learners' autonomous learning ability and provide corresponding support for personalized learning^9^. The combination of AIT and a mobile platform (MP) is more widely applied in modern education, and its influence on higher art education continues to deepen^10^. The AIT and MP are combined to study oil painting teaching.

This study aims to solve some problems existing in higher art education, one of which is the limitation of existing methods in oil painting teaching design. The traditional oil painting teaching mode often lacks the characteristics of individuality and intelligence, and cannot fully meet the learning needs and creative potential of different students. In addition, the existing oil painting image classification methods usually only rely on traditional image features, such as color and texture, ignoring the importance of brush features in oil painting. Given the problems and challenges existing in the current higher art education, such as the lack of students' independent exploration ability and learning interest, the traditional oil painting teaching mode has been unable to meet the demands of modern students. With the rapid development of mobile network technology, smartphones, and other mobile terminals have become an indispensable part of people's daily lives, and the combination of mobile platforms and AIT can bring great potential to higher art education. In painting classification, the selection and classification of image features are closely linked to the deep learning (DL) method. DL is usually independent learning of the features of painted images and classification according to the features. General classification algorithm models cover the decision tree (DT), Naive Bayes, Convolutional Neural Network (CNN), K-Nearest Neighbor (KNN), and so on. Among them, the DL algorithm has been widely used because of the rise of artificial neural networks. The core idea of the DL algorithm is to learn many useful features by training massive data, thus improving the classification accuracy through these features. In recent years, in the field of painting image processing, the DL algorithm with remarkable classification performance has achieved excellent results by learning the features of painting images. According to the personalized learning method to improve oil painting teaching, DL technology is introduced to explore the classification of oil painting. Since the color and brush features of oil painting are vital contents in oil painting learning, this study takes the two features as the background to conduct an intelligent classification of oil painting images. The classification technology is applied in the AI-guided personalized learning mode to enhance the intelligence of the oil painting teaching system. The innovation of this study lies in the combination of traditional oil painting teaching design and MP technology and puts forward a novel teaching mode. Although methods such as Laplace Operator, expansion, and erosion are traditional, this study extends the boundaries of traditional teaching models. The use of MP can provide a more flexible and convenient learning experience, allowing learners to learn oil painting anytime and anywhere, greatly improving the convenience and accessibility of learning. The structure of this study includes the following. Firstly, this study elaborates on the specific content and methods through the introduction of MP-based oil painting teaching design and oil painting classification based on multiple features. Secondly, the construction process, experimental data, and environment of the MP-based personalized painting teaching system are described in detail. Thirdly, the results of oil painting teaching design are investigated, encompassing the results of oil painting classification and the effect of a personalized teaching system based on AIT. Finally, in the “Discussion” and “Conclusion” section, the research results are summarized, and the future research direction and improvement suggestions are put forward. This study aims to explore how to use MP and AIT to improve the teaching mode of oil painting in higher art education to adapt to the learning needs and habits of modern students. By combining the DL algorithm and oil painting feature extraction technology, it is expected that an intelligent oil painting teaching system can be designed, that can offer customized teaching content and feedback according to students' individual needs and learning progress. The results of this study provide a reference for the innovation of teaching mode in higher art education and also afford enlightenment for the application of MP and AIT in other disciplines. The main contributions of this study are as follows:

- Combining AIT and individualized education theory, a personalized oil painting teaching framework based on MP is proposed.
- An oil painting classification model based on CNN, mathematical morphology, and support vector machine (SVM) is constructed, which markedly improves the classification accuracy by extracting brush strokes and color features. Through the analysis of experimental data and performance evaluation, the proposed model’s validity and superiority in oil painting classification are proved.
- This study discusses the application of MP in higher art education and puts forward a flexible and convenient learning mode, which facilitates improving students' learning experience and efficiency.

The structure of the following sections is as follows. “Literature review” section describes in detail the design of personalized oil painting teaching based on MP and the oil painting classification model’s construction method. “Oil painting teaching design” section introduces the experimental environment, data, and pretreatment process, and evaluates the classification model’s performance. “Results analysis of oil painting teaching design on account of MP” section discusses the design and application of a personalized oil painting teaching system. “Conclusion” section 5 summarizes the research results and puts forward the future research direction and improvement suggestions.

## Literature review

The first is a review of the discussions on individualized education and AI. In the discussion on individualized education, American scholar Carroll A. W. explained modern individualized education. He believed that individualized education achieved a balance between the learning environment and the personality characteristics of learners. It was a reasonable match between the learner's personality characteristics and the concepts, knowledge, behavior style, learning environment, acquired skills, and incentive system in a continuous process^11^. A personalized teaching model based on individualized education differed from the traditional unified teaching model. The personalized model emphasized the release of learners' nature to a certain extent, eliminated the shackles of the traditional learning model framework, and advocated that learners take the initiative to fit in with the actual learning environment^12^. Nowadays, AIT and individualized education are applied in the field of English learning. AIT and individualized education have been applied in the domain of English learning. For example, Lexile Framework, Star Reading (STAR), and Accelerated Reader (AR) can accurately test students' reading levels and help students choose reading materials suitable for their English level^13^. In addition, both of them also have certain applications in the study of science curricula. For instance, "Experimental Club", a virtual simulation laboratory, provides customized learning paths for students through the development of cognitive tools. As a result, the student’s analytical and diagnostic capability, innovation consciousness, and operation ability can be improved during the experiment^14^.

The second is a related discussion on brush features. Brushstrokes are the marks left by each stroke as an artist paint. In the study of brush features, scholars conducted a lot of analysis. Jiang et al. automatically extracted Van Gogh's brushstrokes and divided brush features into independent features of geometric creation and interactive features dependent on adjacent distribution, proving that Van Gogh's brushstrokes had strong rhythmic patterns^15^. There were also many studies in the oil painting classification field. For example, Ulicny et al. proposed a classification model integrating various visual features for oil painting^16^. Jayachitra and Prasanth explored the classification of portrait miniatures under various types of brush features^17^. Rostami and Kaveh classified different styles of painting based on the painting features and combined them with an SVM^18^. With the combined development of DL technology and brush features, Knudson and Gupta introduced Fisher information distance based on a Hidden Markov Tree model in the wavelet domain to calculate the style similarity between brushstroke samples and reached an 85% classification accuracy^19^.

In the study of painting classification based on DL, Gupta and Bajaj presented a method of target segmentation by DL, which improved the accuracy of dynamic detection. Through this method, the region-based DL method achieved the most accurate target detection accuracy^20^, and other researchers referred to this method to greatly improve the speed of network training and testing^21^. Then, CNN made a series of breakthroughs in areas such as image classification. Gao et al. proposed a method combining CNN and DT C4.5 classifiers to distinguish different painting styles. Furthermore, according to the bottom features of the image, such as autocorrelation texture features and the newly proposed edge size histogram, bottom features were input into the SVM to classify different painting styles^22^. Papoutsis et al. classified Multi-Layer Perceptron (MLP) and SVM in the fusion method, and the classification accuracy was more than 70%^23^. Krentzel et al. developed a five-layer neural network and showed accurate classification performance by the Imagenet model^24^. With the wide application of CNN in image processing, many researchers made good progress in classification accuracy by image features. Ali combined CNN with color and texture features to design a realistic system of painting^25^. Arco et al. proposed a fusion method of different vector codes, which indicated that typical local features were effective in image recognition and achieved remarkable performance^26^.

The specific pieces of literature involved in the above relevant studies are exhibited in Table 1:

**Table 1:** Comparison of relevant literature.

Year	Author	Advantages	Disadvantages
2021	Jiang et al.	Brush features were automatically extracted, demonstrating a strong rhythmic pattern	The application of DL technology was not involved
2021	Jayachitra and Prasanth	The classification of portrait miniatures under different brush features was explored	DL techniques were not mentioned
2021	Rostami and Kaveh	Painting style classification based on brush features and SVM	DL technology was not involved
2022	Ulicny et al.	A classification model integrating various visual features was proposed	DL technology was not involved
2022	Knudson and Gupta	Style similarity calculation based on Fisher information distance	Specific DL techniques were not mentioned
2023	Gupta and Bajaj	DL object segmentation method was proposed to improve the accuracy of dynamic detection	Other DL techniques were not involved
2023	Gao et al.	CNN and DT C4.5 classifiers were combined to distinguish diverse painting styles	Other DL techniques were not involved
2023	Papoutsis et al.	In the fusion method of MLP and SVM, the classification accuracy was more than 70%	Other DL techniques were not involved
2023	Krentzel et al.	The accurate classification performance was demonstrated based on the Imagenet model	Other DL techniques were not involved
2023	Ali	A realistic painting system was designed by integrating CNN with color and texture features	Other DL techniques were not involved
2023	Arco et al.	A fusion method of various vector codes was proposed, and remarkable performance was achieved	Specific DL techniques were not covered

On account of the above content, it can be found that the combination of personalized models and AI has a positive impact on students' learning. However, there are few pieces of research on the personalized model in the field of painting, so this study combines AIT with this kind of model and applies it to the teaching of oil painting. Many scholars have studied oil painting classification based on the DL method and proved that brush features are one of the important characteristics of this classification. However, the existing research principally analyzes the brush features of different painters, and there are few studies on the classification of diverse schools of oil painting. Thereupon, the classification of oil painting is studied on account of brush features and different schools. Besides, it is applied in an AI-guided personalized teaching mode and strives to improve the current teaching mode and efficiency of oil painting teaching.

## Oil painting teaching design

### Design of extraction method

The ends of the brushstrokes tend to form more distinctive tracks that appear on the edges of the image. Image edge refers to the local area where the brightness visible to the naked eye changes and the pixel mutation occurs^27^. Edge detection methods usually consist of various operators. Image edges of paintings generally include three forms: mutant, slow, and linear^28^. Among them, mutant refers to the edge of the image where the mutation of pixel value occurs in a certain area. Slow type signifies that the gray value (GV) of pixel points keeps stable for some time, then suddenly changes at some point, and then tends to be stable. Linear edge means the edge where the GV changes rapidly and restores the original GV in a short time. Based on this, the first hypothesis is proposed. Compared with the color feature, the brush feature is more important in image classification.

Current edge detection methods can be divided into edge detection by the first-order and second-order differential operation^29^. Among them, the Canny Operator and Laplace Operator are commonly used in the second-order differential operation. The algorithm steps of the Canny Operator cover removing noise, finding gradient value, and detecting edge.

The Laplace Operator is defined as Eq. (1):

1 $$\Delta f={\nabla }^{2}f=\nabla \cdot \nabla f$$

$\nabla f$ refers to the gradient; $\nabla \cdot f$ stands for divergence.

In recent years, mathematical morphology has been widely applied in machine vision research. It is an image analysis subject based on lattice theory and topology, serving as the foundational theory for mathematical morphology image processing. The fundamental operations encompass opening & closing operations, erosion and dilation, skeleton extraction, limiting erosion, hit-and-miss transformation, morphological gradient, Top-hat transformation, particle analysis, watershed transformation, etc. The scope of research in mathematical morphology spans the design of element composition, the exploration of a morphological algorithm, the creation of the improved filter, the research of dynamic things, the processing of rich information, the implementation of optical hardware, the research of nonlinear wavelet, the exploration of multi-resolution signal, and the optimization algorithm^30^.

The current trend of morphological operation is to improve its common usage and enhance its practicability. Common operations in morphological operations involve dilation, erosion, closing, and opening^31^. The erosion operation can be written as Eq. (2):

2 $$A\ominus B=\left\{x,y\mid (B{)}_{xy}\subseteq A\right\}$$

*A* expresses the image set; *B* refers to the binary element in the set; *x* and* y* represent the image’s pixel. Equation (2) indicates that structure B is used to erode A.

Dilation can "enlarge" the range of the target area, merging background points in contact with the area into the target, and expanding the target boundary outside. Its function is to fill some holes in the target region and eliminate small particle noise contained in the region^32^. The dilation operation is expressed in Eq. (3).

3 $$A\oplus B=\left\{x,y\mid (B{)}_{xy}\cap A\ne {\varnothing }\right\}$$

Equation (3) means that structure *B* is adopted to dilate *A*, and the origin of structure element *B* is translated to the position of the image pixel (*x*, *y*). If the intersection of *B* and *A* at (x, y) is not empty (that is, at least one of the image values corresponding to A at the position of element 1 in B is 1), then the x corresponding to the output image is assigned a value of 1. Otherwise, it is assigned a value of 0.

The definitions of opening operation ($A\circ B$) and closing operation ($A\cdot B$) are as follows.

4 $$A\circ B=A\ominus B\oplus B$$

5 $$A\cdot B=A\oplus B\ominus B$$

The opening operation is to perform the erosion operation first, and then the division operation; The opposite is true for closing operations.

Compared with other operators, the Sobel operator has a larger filter size and better anti-noise. Thus, the edge detection method in this study uses the Sobel algorithm, which is applied to the gray image to recognize edge pixels^33^. The Sobel operator belongs to the edge detection algorithm based on the first-order gradient algorithm. In the process of brushstroke extraction, the convolution kernel is shown in Eqs. (6) and (7):

6 $${G}_{X}=\left[\begin{array}{ccc}-1& 0& +1\\ -2& 0& +2\\ -1& 0& +1\end{array}\right]$$

7 $${G}_{Y}=\left[\begin{array}{ccc}-1& -2& -1\\ 0& 0& 0\\ +1& +2& +1\end{array}\right]$$

${G}_{X}$ and ${G}_{Y}$ represent the convolution kernel; *X* and *Y* display directions of the coordinate axis.

The mathematical expressions of Sobel operators read:

8 $$\begin{aligned}{G}_{x} & ={f}_{x}(x,y)=f(x-1,y+1)+2f(x,y+1)+f(x+1,y+1) \\ & \quad - f(x-1,y-1)-2f(x,y-1)-f(x+1,y-1)\end{aligned}$$

9 $$\begin{aligned}{G}_{y} &={f}_{y}(x,y)=f(x+1,y-1)+2f(x+1,y)+f(x+1.y+1) \\ & \quad - f(x-1,y-1)-2f(x-1,y)-f(x-1,y+1)\end{aligned}$$

*f* refers to the function operation for *x* and *y*.

The matrix form of the Sobel operator is illustrated in Eq. (10) and Eq. (11):

10 $${G}_{x}={f}_{x}(x,y)=\left[\begin{array}{ccc}-1& 0& 1\\ -2& 0& 2\\ -1& 0& 1\end{array}\right]$$

11 $${G}_{y}={f}_{y}(x,y)=\left[\begin{array}{ccc}-1& -2& -1\\ 0& 0& 0\\ 1& 2& 1\end{array}\right]$$

When the GV of pixels on the oil painting image changes in gradient, the change of the GV is the GV of each pixel in the image combined vertically and horizontally. The calculation method is implied in Eq. (12):

12 $$G=\sqrt{{G}_{x}^{2}+{G}_{y}^{2}}$$

In the process of brush feature extraction, the oil painting image is first converted into a gray image. Then the Sobel operator is used for operation combined with the 3*3 filter. Additionally, the image and filter are convolved to get the gradient image.

Compared with other operators, the Sobel operator’s filter size is larger, and its anti-mania properties are more significant. Regarding painting images, the grayscale change values of target and neighborhood pixels within a certain range are more sensitive, which can better grasp details and better reflect the shape and characteristics of brushstrokes. However, after brush feature extraction by the Sobel operator, there are still problems such as image edge fracture or a rather messy edge line prominence. For example, an edge line detected from an image might not be a single brushstroke. In a certain area of the image edge, some relatively complex edge lines are also highlighted. The edge line around the brushstrokes may not be completely sharp and thus break during detection, which is the problem above. In this study, the morphological operation method is employed to solve these two problems. After performing edge detection operations, some brushstrokes may not be fully detected due to a lack of sharpness around the edge lines. The opening of the morphological operation can smooth the outline of the edge of the painting and eliminate the fine brushstrokes in the edge area. Next, the closing operation connects the eliminated brush edge line and fills in the fracture. Thereby, the operation of opening first and then closing is adopted for brushstroke processing at the edge^34^.

**Figure 1:**
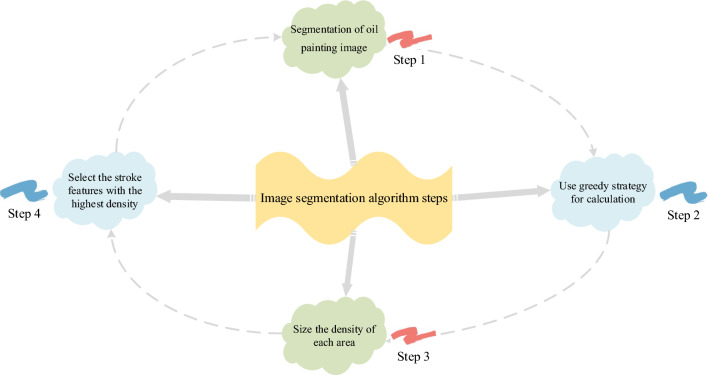
The steps of the image segmentation algorithm.

Here, a representative brush feature is selected for classification input. The steps of the image segmentation algorithm are denoted in Fig. 1.

**Figure 2:**
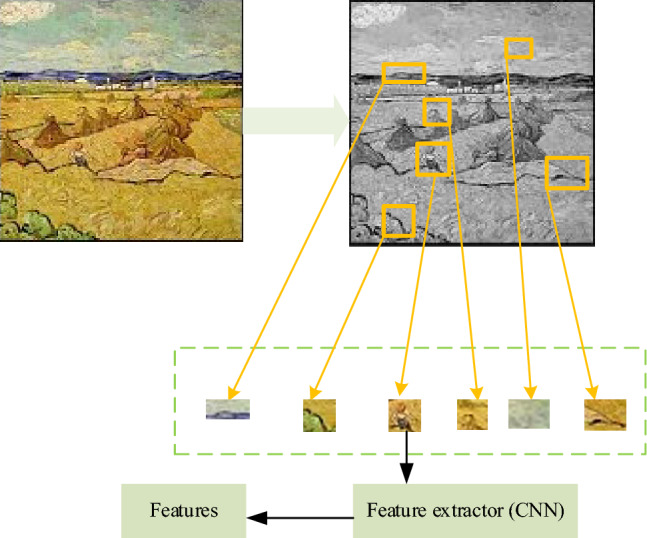
The rendering process of representative brushstrokes.

Figure 1 illustrates the image segmentation method, which comprises four distinct steps. In step 1, the oil painting image undergoes morphological manipulation and is partitioned into 2,000 smaller components. In step 2, the greedy strategy is used to calculate the gradient similarity value between adjacent regions of each pair of sub-painting images, facilitating the merging of the two sub-painting images with the closest proximity. Step 3 involves sorting each region based on its density, while Step 4 entails selecting the brush feature that ranks among the top 6 in density. Ultimately, the brush feature with the top 6 densities is selected as the representative brush strokes of this painting. The rendering process of representative brushstrokes is plotted in Fig. 2.

### Oil painting classification based on multiple features

**Figure 3:**
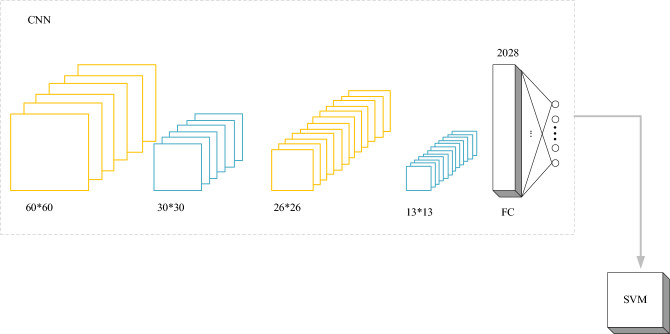
The architecture of the CNN model.

A brush features-based CNN model is designed. Following the morphological operation and segmentation extraction of the image subsequent to edge detection, the resulting image is employed as input for CNN (size 64 × 64) for learning and training purposes. The CNN model consists of 5 layers, as demonstrated in Fig. 3.

In Fig. 3, the C1 layer utilizes six 5 × 5 cores to filter the input data and generate six 60 × 60 mappings. Subsequently, the S1 layer employs a sub-sampling rate of 2, performing maximum pooling layer operations on each mapping to reduce the feature size and model parameters. The C2 layer and the second convolutional layer use 12 5 × 5 kernels to filter the data operation, resulting in 12 26 × 26 mappings. The S2 layer continues to reduce the feature size. By designing the fully connected layer, 2,028-dimensional vectors are obtained, and ultimately, 1,014-dimensional features are output.

**Figure 4:**
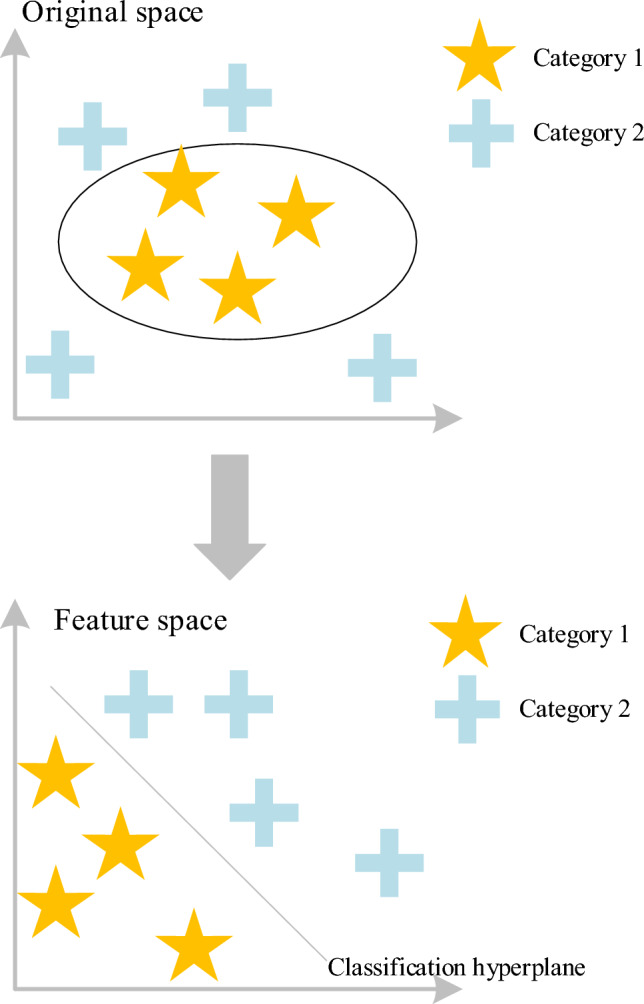
The basic principle of the SVM model.

SVM is a kind of generalized linear classifier for the binary classification of data in supervised learning scenarios. Its decision boundary represents the maximum margin hyperplane for solving learning samples, transforming the problem into convex quadratic programming. Compared with logistic regression and neural networks, SVM offers a more transparent and potent method for learning complex nonlinear equations. The SVM model is usually used in the field of image processing. The basic principle of the model is presented in Fig. 4.

SVM maps vectors to a higher dimensional space, establishing a hyperplane with a maximum margin. Two parallel hyperplanes are constructed on either side of the hyperplane separating data. The maximization of distance between these parallel hyperplanes is prioritized for optimal separation. The hypothesis posited is that a larger distance or gap between parallel hyperplanes corresponds to a reduced total error of the classifier. Consequently, the second hypothesis asserts that SVM exhibits superior performance in the context of image classification.

The basic calculation of SVM is signified in Eq. (13):

13 $$f(x)=\sum_{i=1}^{n} {w}_{i}k\left(x,{x}_{i}\right)+b$$

*w* and *b* refer to two parameters of hyperplane; *w* stands for the normal vector of the vector; *i* is the serial number; *n* represents *n*-dimensional feature space; $k\left(x,{x}_{i}\right)$ displays the kernel function; *x* means an argument.

SVM is to find the optimal hyperplane in a binary classification problem, and the hyperplane can be expressed as Eq. (14):

14 $$f(x)={w}^{T}\text{x}+b$$

The objective function of the kernel function in SVM is as follows:

15 $$\underset{w,\varepsilon ,b}{min} \left\{\frac{1}{2}\parallel w{\parallel }^{2}+c\sum_{i=1}^{n} {\varepsilon }_{i}\right\}$$

*c* denotes the regularization parameter; $\varepsilon$ means slack variable.

Lagrange multipliers are introduced to reconstruct the objective function through quadratic programming, as exhibited in Eq. (16):

16 $$\begin{array}{c}max\sum_{i=1}^{m} {\lambda }_{i}-\frac{1}{2}\sum_{i,i=1}^{m} {\lambda }_{i}{\lambda }_{i}{y}_{i}{y}_{j}k\left({x}_{i},{x}_{j}\right)\\ 0\le {\lambda }_{i}\le C,i=\text{1,2},3\dots m,\sum_{i=1}^{m} {\lambda }_{i}{y}_{i}=0\end{array}$$

$\lambda$ stands for the Lagrange multiplier; $m$ expresses the number of training data; $y$ represents the label of the painting image; $x$ refers to its brush feature representation.

**Figure 5:**
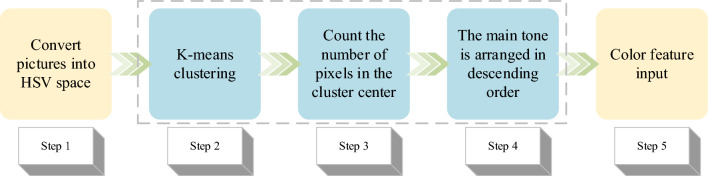
Classification process of color feature.

This study divides the oil painting dataset into different genres, including Cubism, Renaissance, Baroque, Rococo, and Impressionism. The color feature is one of the important features of oil painting. Hence, this study also combines the color features of oil paintings for classification, which can be classified into five steps, as represented in Fig. 5.

The first step involves converting oil paintings into the Hue Saturation Value (HSV) model, encompassing hue, saturation, and brightness attributes of oil paintings^35^. In the second step, the K-means clustering method is implemented in the HSV model, and the generated colors are quantized in space. In the third step, 20 colors with a value of 20 are randomly selected and employed for 20 clusters. The mean value of each class of colors is determined, and this process iterates until color stability is achieved, terminating the algorithm. Ultimately, 20 clustering centers are formed. In the fourth step, according to the number of pixels in the center point of K-means clustering, the colors are sorted from largest to smallest, and the top 6 are taken as the main colors of input features. In the fifth step, the color features and brush features extracted by the CNN are input into SVM together.

**Figure 6:**
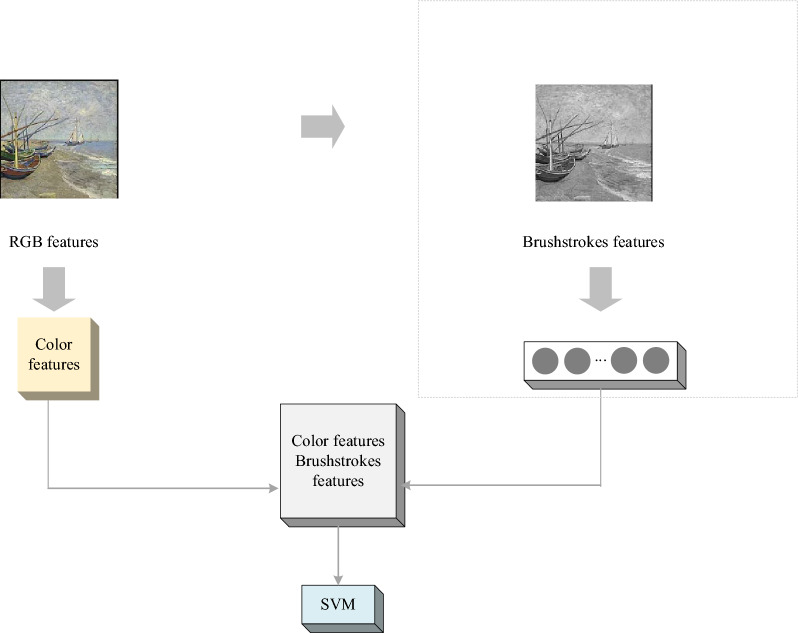
Classification flow of double feature extraction.

The separate color feature and the relevant feature combined with the brush features are employed to conduct the classification and comparison experiment of oil painting. The experimental process is portrayed in Fig. 6.

### Construction of personalized painting teaching system by MP

With the swift growth of MPs such as iPads and mobile phones, the current education system is developed on the MPs. The personalized teaching system of intelligent oil painting constructed here also faces the challenge of MP. The construction of this teaching system combines AIT, collaborative filtering (CF) system, big data analysis (BDA) technology, and oil painting classification technology mentioned above. The core content includes a painting creation module, a personal center, a community module, and a painting auxiliary module^36^.

The community module covers the selection of material learning content. This part applies the oil painting classification technology designed in this study to classify oil paintings according to diverse factions, thus improving the retrieval and learning efficiency of oil painting types in learning. Furthermore, it also encompasses live and video teaching. The painting creation module offers drawing tools and painting capabilities so that various templates can be used for painting. The painting auxiliary module involves intelligent recommendation, auxiliary tools, intelligent recognition, evaluation feedback, real-time auxiliary function, flow-process diagram generation, etc., which helps better complete personalized painting creation^37^. The main contents of the personal center include a painting record, a collection, and so on. Among them, the CF system provides an intelligent push for oil paintings of related genres according to different search preferences.

### Experimental dataset

The dataset used in this study was obtained from the online collection platform (https://gallerix.asia/a1/). The dataset comprises 1000 oil paintings from five different art styles (Baroque, Cubism, Impressionism, Renaissance, Rococo), with each style containing 200 artworks. During the dataset construction process, carefully selected representative oil paintings were chosen to ensure coverage of typical characteristics and stylistic differences in each style.

In the experiment, the dataset was divided into training and testing sets in a 75% to 25% ratio. Specifically, the training set for each art style consisted of 150 oil paintings, used for model training and parameter optimization. The testing set included 50 oil paintings for each style, used to evaluate the model's performance on unseen data. This partitioning strategy aims to ensure that the model receives sufficient training and testing across various art styles, allowing for a more comprehensive assessment of its performance in the oil painting style classification task.

### Experimental platform

The experimental environment is outlined in Table 2.

**Table 2:** The experimental environment.

Item	Processor/operating system	Memory/development environment	Install type
Hardware environment	Intel Core i7-7700 Central Processing Unit (CPU) @3.60 GHz	8.00Gigabyte (GB)	64-bit operating system
Software environment	Windows 10 Home Edition	Pycharm 2019.2 IDE (Integrated Drive Electronics) Community Edition	

In this study, a computer with Intel Core i7-7700 CPU and 8 GB memory is selected as the hardware environment to ensure the stability and efficiency of the experiment. At the same time, a 64-bit operating system is employed to make full use of computing resources. In the software, Windows 10 Home Edition is chosen as the operating system and Pycharm 2019.2 IDE (Community Edition) is used as the integrated development environment. Based on their stability, ease of use, and wide applicability, these choices can effectively support related research work.

### Hyperparameter setting

This study’s hyperparameter settings are shown in Table 3:

**Table 3:** Hyperparameter setting.

Hyperparameter	Value
Learning rate	0.001, 0.005, 0.01
Batch size	32, 64, 128
Optimizer	Adam, SGD
Activation function	ReLU, Sigmoid
Initial weight	Random initialization, Xavier initialization
Epochs	50, 100, 150
Regularization parameters	L2 regularization, λ = 0.01
Hyperparameter	Value
Learning rate	0.001, 0.005, 0.01

In model training, hyperparameter settings have a significant impact on the final performance. The learning rate is adjusted among 0.001, 0.005, and 0.01 to balance the model's convergence speed and stability. Batch sizes are set at 32, 64, and 128 to explore the impact of different data batches on training efficiency and model accuracy. The Adam and SGD optimizers are employed, each with its unique convergence properties and suitable scenarios. Activation functions ReLU and Sigmoid are chosen to test their performance in handling nonlinear relationships. Initial weights are set using random initialization and Xavier initialization to ensure a reasonable weight distribution at the start of training. Training epochs are set at 50, 100, and 150 to fully evaluate the model's performance over diverse training periods. Regularization parameter L2 is used with λ = 0.01 to prevent overfitting and improve generalization ability.

## Results analysis of oil painting teaching design on account of MP

### Learning rate analysis

**Figure 7:**
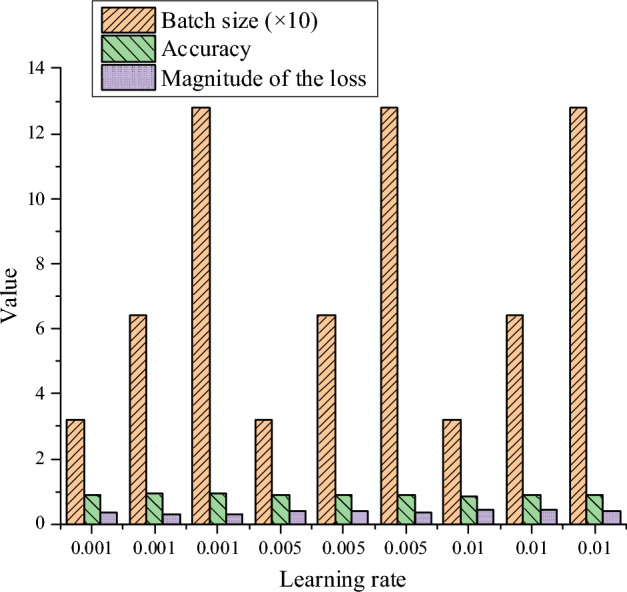
Setting of learning rate.

The data performance of the learning rate under different batch sizes is revealed in Fig. 7:

In Fig. 7, the learning rate notably affects the model's accuracy and loss value. When the learning rate is 0.001, the model achieves high accuracy and low loss values across all batch sizes, with the best performance at a batch size of 128, reaching an accuracy of 92.50% and a loss value of 0.28. However, when the learning rate increases to 0.01, the model's accuracy drops significantly, with a maximum of only 89.00%, and the loss value increases correspondingly. This indicates that an excessively high learning rate may cause the model to overshoot the optimal solution, affecting convergence. The choice of batch size also impacts model performance. When the learning rate is fixed, increasing the batch size generally improves accuracy and reduces loss values. For instance, when the learning rate is 0.001, the accuracy of batch sizes 32, 64, and 128 is 90.25%, 91.75%, and 92.50%, respectively, with a corresponding decrease in loss values. This improvement is likely because larger batch sizes provide more stable gradient estimates, aiding in better model training and convergence. Overall, with a learning rate of 0.001 and a batch size of 128, the model performs best, achieving the highest accuracy and the lowest loss value. This suggests that under the settings of this study, a learning rate of 0.001 and a larger batch size are the optimal hyperparameter choices. These results offer vital insights for subsequent model optimization and application, indicating that using a learning rate of 0.001 and a batch size of 128 can yield the best performance in practical applications.

### Analysis of oil painting classification results according to color and brush features

**Figure 8:**
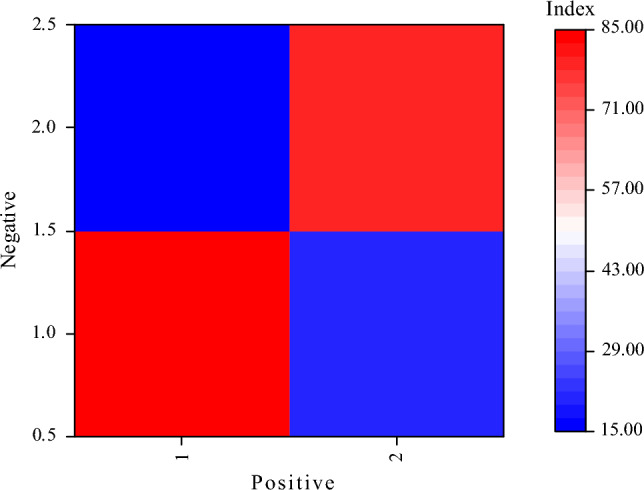
Confusion matrix result.

In this study, the performance of the MP-based oil painting teaching design is evaluated through experiments. Based on the experimental results, the following confusion matrix is obtained to describe the model’s classification. True Positive (TP): The number of samples that the model correctly identifies as an oil painting teaching design. True Negative (TN): The number of samples that the model correctly identifies as non-oil painting teaching designs. False Positive (FP): The model incorrectly identifies the samples of the non-oil painting teaching design as the number of samples of the oil painting teaching design. False Negative (FN): The model incorrectly identifies the number of samples of the oil painting teaching design as the number of samples of the non-oil painting teaching design. The data is depicted in Fig. 8:

Based on Fig. 8, the indicators can be calculated. TP: The number of samples that the model correctly predicts as positive is 85. TN: The number of samples correctly predicted as negative by the model is 80. FP: The model incorrectly predicts the number of samples of the negatives as positive, which is 15. FN: The model incorrectly predicts the number of samples of the positives as the number of samples of the negatives, which is 20. Based on the results of the confusion matrix, the model’s performance can be considered relatively good.

**Figure 9:**
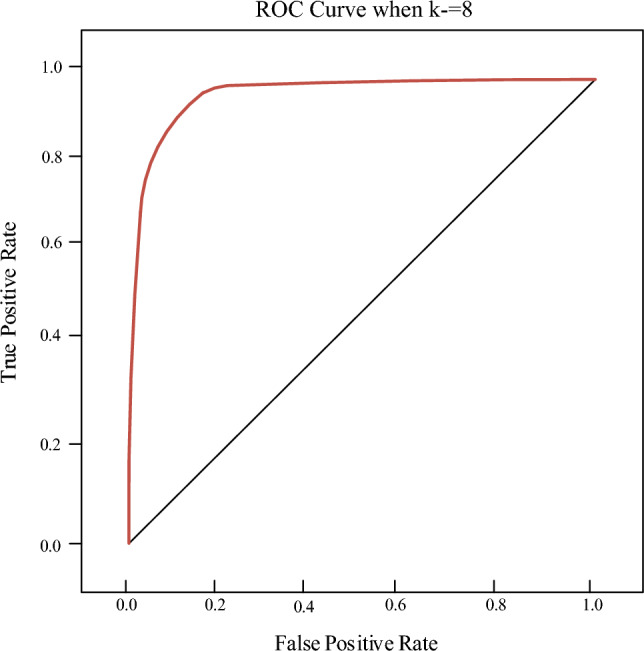
AUC plot.

In the area under the curve (AUC) plot, as shown in Fig. 9, the parameter k represents the number of categories for the model. After comparison, in this study, the value of k is set to 8, indicating the presence of 8 different categories of samples. The AUC plot illustrates the evaluation results of the performance of oil painting teaching design based on a mobile platform. The horizontal axis represents the False Positive Rate, and the vertical axis represents the True Positive Rate. The AUC, reflecting the performance of the model classifier, is 0.95, illustrating that the model performs well in multi-class classification tasks with high accuracy.

**Figure 10:**
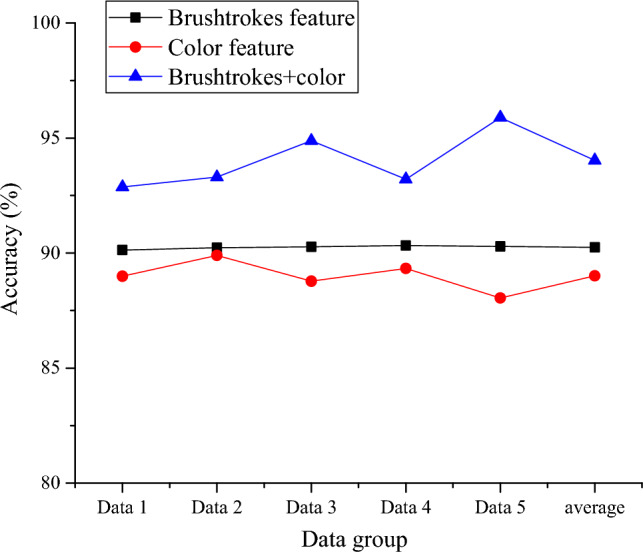
Accuracy of painting classification under single-feature and the double feature.

In this study, oil paintings in the test set are divided into 5 equal parts (represented by Data 1 to Data 5 in Fig. 10), and the classification accuracy under a single feature and the double feature is tested. The results are revealed in Fig. 10.

Figure 10 refers that when the brush feature and color feature are extracted, the average classification accuracy (ACA) is about 90.25% and more than 89%, respectively. When extracting the two features, the classification model’s ACA for oil painting reaches 94.03%. In the case of the same classification model, the quality of brushstrokes has a significant impact on the final experimental results. The classification accuracy based on brush features is higher than that without brush features. Therefore, the first hypothesis is true.

**Figure 11:**
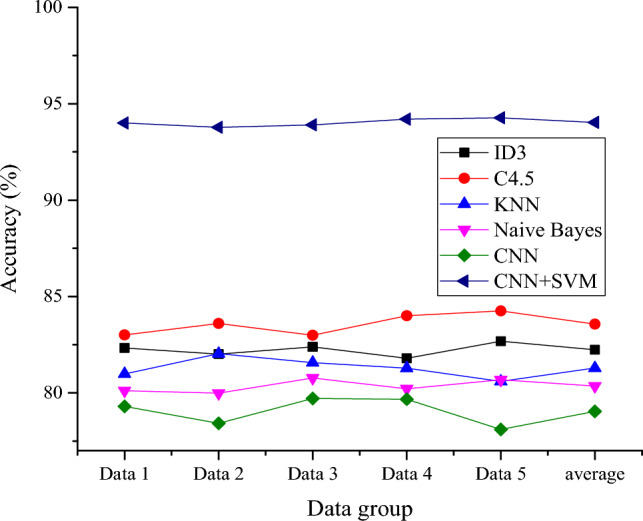
Comparison of classification accuracy of different classifiers under color features.

After the brush feature is learned and trained by CNN, the color features are combined with the input into SVM, and the results are compared with diverse classifiers on the same dataset, as indicated in Fig. 11. The classifiers include Iterative Dichotomiser (ID)3, DT C4.5, Naive Bayes, KNN, and CNN.

In Fig. 11, the ACA of ID3 is 82.24%, and that of DT C4.5 is 83.57%. Both algorithms belong to the DT, so the accuracy rates are close. The ACA of SVM is the best, with 94.03%. Classifiers are often used for prediction in different application fields. Each classifier has its characteristics, and diverse classifiers are suitable for different situations. To sum up, in the process of studying and analyzing painting images based on brush features, SVM has a more significant effect. Thus, the second hypothesis holds.

**Figure 12:**
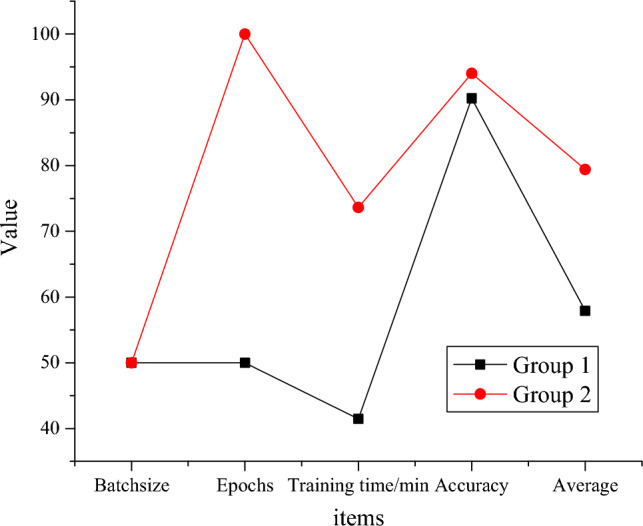
Accuracy and training time based on brush features under various CNN parameters.

Diverse network parameters have an impact on classification accuracy and time. The training time and accuracy based on brush features under various CNN parameters are compared, and the results are described in Fig. 12.

Figure 12 details that the speed of training with epochs data of size 50 is faster than that of epochs raw data of size 100, but the accuracy is slightly reduced. When comparing the differences between diverse data sizes in terms of training time and accuracy, the data size is often determined by the original pixel subset. Better accuracy can be obtained by inputting data of diverse sizes into CNN. During the model training process, using a smaller batch size (50) can enhance training speed since it requires fewer computations. However, despite the faster training speed, there is a slight decrease in the training accuracy due to the smaller dataset. This indicates a trade-off during the training process: a balance needs to be struck between training speed and accuracy. Additionally, in practical applications, selecting an appropriate dataset size is crucial for achieving better model performance. Typically, the dataset size depends on the size of the original pixel subset, and choosing different sizes for data input into the CNN can influence the model's accuracy to some extent. Therefore, by carefully selecting and optimizing the training dataset, it is possible to improve training efficiency while ensuring model accuracy.

### Personalized drawing system analysis combined with AIT

**Figure 13:**
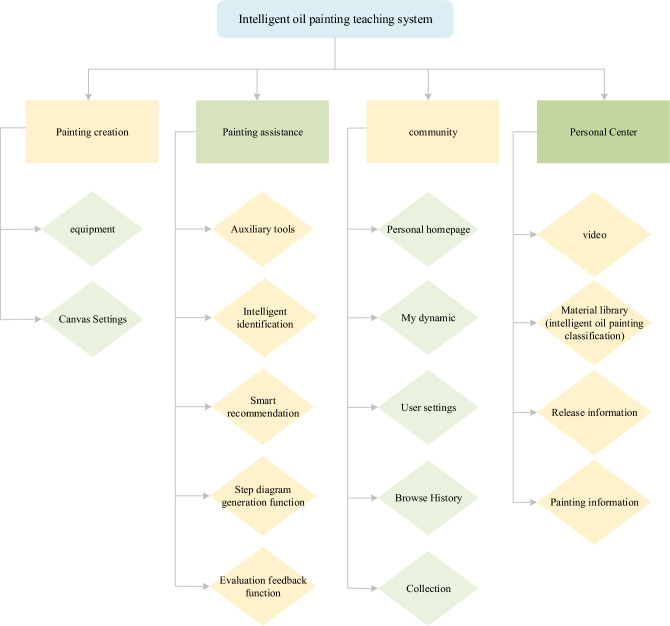
The architecture of the intelligent oil painting teaching system.

On the strength of different oil painting styles, combined with BDA, AIT, and oil painting classification technology, the intelligent oil painting teaching system is constructed. Figure 13 demonstrates the basic structure.

Given the content in Fig. 13, the teaching system helps to improve the oil painting’s intelligent classification level and enhance learning efficiency. The video teaching module in the personalized system also has a positive effect on teaching efficiency. The intelligent learning spaces composed of an MP, learning resources, learning tools, intelligent hardware resources, etc. can improve the learning effect of oil painting in higher art education.

Taking the actual oil painting teaching scene in universities as an example, the proposed model serves as the foundation for an individualized education approach coupled with real-time one-on-one support tailored to individual needs. This facilitates the creation of a user-friendly online oil painting teaching system. The real-time intelligent auxiliary and step diagram generation functions are designed and developed. The real-time intelligent assistance function primarily captures students' interactions with painting tools, color choices, and ongoing painting steps. The system provides suggestions on line modification or color use that it thinks is more suitable for the painting, corrects step errors in the painting process, and offers relevant reference videos or excellent artworks matching the learner's painting style. In this process, the teacher plays a guiding role, answering students' drawing questions online according to their learning contexts. It enables learners to quickly master painting skills, and improve their creative ability in the field of painting that they are interested in. Students' painting potential is fully tapped to help learners engage in painting creation more easily, realize intelligent auxiliary and interaction, and achieve the teaching goal of personalized teaching. Personalized evaluation is rooted in individualized content and forms the foundational premise for the implementation of personalized teaching. This part of the evaluation is completed by teachers online. To sum up, the practical implementation of the proposed model has the potential to enhance the status quo of art education. Combined with "Internet+", the intelligent learning service system of individualized education is constructed. While prioritizing cutting-edge learning technologies, the educational philosophy of "student-centered" is actualized. Intelligent learning environment is a student-centered intelligent learning space comprising intelligent hardware resources, learning tools, learning resources, teachers, and students. This learning space facilitates students' seamless interaction with learning resources or systems at any time and from any location, offering personalized learning guidance and advice. Centering on the concept of "teaching students according to their aptitude", the aim is to provide students with better learning services through intelligent technology, thereby fully exploiting their full creative potential. This approach represents a crucial research direction in the contemporary realm of personalized painting teaching methodologies.

## Discussion

Painting is an aspect of image processing. With the increasing number of painted images, researchers are increasingly involved in the effective processing of such images, focusing on the extraction of elements like strokes, textures, white spaces, color features, etc. Among them, the brush feature is an important part of the painting image. This study presents a method to classify Western paintings by combining the brush and color features. First, the original RGB image of each Western image is converted into the HSV model. Subsequently, the k-means clustering method is performed on the next HSV model. The K-value is ordered by the central point of the digital k-means clustering method in descending order, and the ordered colors are included in the main tone of the color input feature. A comparison between color and painting features against monochrome features demonstrates the substantial importance of brush features in painting classification studies. The intelligent classification of oil painting is studied based on CNN. Among them, the use of brush features greatly influences the experimental results for its classification. The classification accuracy of the brush feature is higher than that of the color feature. When double-feature is used for classification, that is, two features are combined, the classification accuracy is the highest, exceeding 94%. Compared with the research on oil painting by Qin^38^ and Zhu^39^ using the AI method, it is found that its classification accuracy is basically consistent with the accuracy in this study. Different classifiers are suitable for diverse situations in the experiments of various classifiers, and SVM is the best here. In this study, the individualized education system designed by AIT is used for MP to provide personalized model and drawing learning, personalized work evaluation, and overall improvement of learning effect.

## Conclusion

Traditional higher art education in China has certain shortcomings. This study establishes an intelligent oil painting image classification system and a personalized intelligent education system based on AI and DL technologies. In this process, the brush feature is used as one of the extraction features for oil painting image classification, which is not common in past studies. A successful oil painting intelligent classification model has been implemented with an accuracy rate of 94.03%. Additionally, an architecture for a personalized oil painting teaching system incorporating advanced technologies has been designed, offering new ideas and approaches for individualized education. Despite achieving certain results, this study has some limitations. Firstly, the relatively small size of the dataset may impose limitations on the generalizability of the results. Secondly, there is still a need to enhance the explanation and application of advanced technologies like AIT in the personalized oil painting teaching system. Furthermore, it is acknowledged that the application effectiveness on an MP has not been comprehensively tested. To address these limitations and improve the study, plans include collecting more oil painting datasets and deepening the explanation and application of AIT in the personalized oil painting teaching system. Emphasis will also be placed on adding application effectiveness to an MP for a more comprehensive evaluation of the system's performance and applicability. Future research will focus on increasing practical case studies and expanding the measurement content of the model to enhance the credibility and practicality of this study and related documentation are provided in the supplementary information.

### Supplementary Information

Supplementary Information.

## Data Availability

All data generated or analyzed during this study are included in this published article [and its supplementary information files]. If someone wants to request the data from this study please contact the Corresponding author (Guodong Yi, 23526933@qq.com).

## References

[1] You Y (2022). Online technologies in dance education (China and worldwide experience). Res. Dance Educ..

[2] Xu L, Zhou Q (2021). App design of distance art education platform under internet ecological environment. Int. J. Electr. Eng. Educ..

[3] Yan ZY (2021). An empirical analysis of the practical value of art practice based on the development of art education concepts. Tob. Regul. Sci..

[4] Yang G (2021). The imagery and abstraction trend of Chinese contemporary oil painting. Linguistics Cult. Rev..

[5] Dignum V (2021). The role and challenges of education for responsible AI. Lond. Rev. Educ..

[6] Yavuz M, Çorbacıoğlu E, Başoğlu AN, Daim TU, Shaygan A (2021). Augmented reality technology adoption: Case of a mobile application in Turkey. Technol. Soc..

[7] Liu Y, Luo J, Zhang L (2021). The effects of mobile payment on consumer behavior. J. Consum. Behav..

[8] Rasulova N, Salieva D (2021). Fuzzy logic in creating adaptive intelligent learning. InterConf..

[9] Qu J (2021). Research on mobile learning in a teaching information service system based on a big data driven environment. Educ. Inf. Technol..

[10] Papadakis S, Kalogiannakis M, Zaranis N (2021). Teaching mathematics with mobile devices and the Realistic Mathematical Education (RME) approach in kindergarten. Adv. Mobile Learn. Educ. Res..

[11] Lim LA, Dawson S, Gašević D, Joksimovića S, Pardo A, Fudge A (2021). Students’ perceptions of, and emotional responses to, personalised learning analytics-based feedback: An exploratory study of four courses. Assess. Eval. High. Educ..

[12] Mousavinasab E, Zarifsanaiey N, NiakanKalhori SR, Rakhshan M, Keikha L, Saeedi MG (2021). Intelligent tutoring systems: A systematic review of characteristics, applications, and evaluation methods. Interact. Learn. Environ..

[13] van Rooij E, Fokkens-Bruinsma M, Jansen E (2021). Factors that influence PhD candidates’ success: The importance of PhD project characteristics. Stud. Contin. Educ..

[14] Chaipidech P, Srisawasdi N, Kajornmanee T, Chaipah K (2022). A personalized learning system-suported professional training model for teachers' TPACK development. Comput. Educ. Artif. Intell..

[15] Jiang W, Wang X, Ren J, Li S, Sun M, Wang Z (2021). MTFFNet: A multi-task feature fusion framework for Chinese painting classification. Cogn. Comput..

[16] Ulicny M, Krylov VA, Dahyot R (2022). Harmonic convolutional networks based on discrete cosine transform. Pattern Recogn..

[17] Jayachitra S, Prasanth A (2021). Multi-feature analysis for automated brain stroke classification using weighted Gaussian naïve Bayes classifier. J. Circuits Syst. Comput..

[18] Rostami O, Kaveh M (2021). Optimal feature selection for SAR image classification using biogeography-based optimization (BBO), artificial bee colony (ABC) and support vector machine (SVM): A combined approach of optimization and machine learning. Comput. Geosci..

[19] Knudson KC, Gupta AS (2022). Assessing cerebellar disorders with wearable inertial sensor data using time-frequency and autoregressive hidden Markov model approaches. Sensors.

[20] Gupta K, Bajaj V (2023). Deep learning models-based CT-scan image classification for automated screening of COVID-19. Biomed. Signal Process. Control.

[21] Rosales, R., Popov, P. & Paulitsch, M. Evaluation of Confidence-based Ensembling in Deep Learning Image Classification, Vol. 13. arXiv preprint arXiv:2303.03185 (2023).

[22] Gao L, Wu Y, Yang T, Zhang X, Zeng Z, Chan CKD (2023). Research on image classification and retrieval using deep learning with attention mechanism on diaspora Chinese architectural heritage in Jiangmen, China. Buildings.

[23] Papoutsis I, Bountos NI, Zavras A, Michail D, Tryfonopoulos C (2023). Benchmarking and scaling of deep learning models for land cover image classification. ISPRS J. Photogramm. Remote Sens..

[24] Krentzel D, Shorte SL, Zimmer C (2023). Deep learning in image-based phenotypic drug discovery. Trends Cell Biol..

[25] Ali YMB (2023). Adversarial attacks on deep learning networks in image classification based on Smell Bees Optimization Algorithm. Future Gener. Comput. Syst..

[26] Arco JE, Ortiz A, Ramírez J, Martínez-Murcia FJ, Zhang YD, Górriz JM (2023). Uncertainty-driven ensembles of multi-scale deep architectures for image classification. Inf. Fusion.

[27] Krithika LB, Priya GG (2021). Graph based feature extraction and hybrid classification approach for facial expression recognition. J. Ambient Intell. Human. Comput..

[28] Sachar S, Kumar A (2021). Survey of feature extraction and classification techniques to identify plant through leaves. Expert Syst. Appl..

[29] Raheja S, Kumar A (2021). Edge detection based on type-1 fuzzy logic and guided smoothening. Evol. Syst..

[30] Debnath B, Sarkar PP (2021). Quantification of random pore features of porous concrete mixes prepared with brick aggregate: An application of stereology and mathematical morphology. Construct. Build. Mater..

[31] Vivone G, D'amico G, Summa D, Lolli S, Amodeo A, Bortoli D (2021). Atmospheric boundary layer height estimation from aerosol lidar: A new approach based on morphological image processing techniques. Atmos. Chem. Phys..

[32] Nogueira K, Chanussot J, Dalla Mura M, Dos Santos JA (2021). An introduction to deep morphological networks. IEEE Access..

[33] Gao P, Song Y, Song M, Qian P, Su Y (2022). Extract nanoporous gold ligaments from SEM images by combining fully convolutional network and Sobel operator edge detection algorithm. Scripta Materialia..

[34] Chetia R, Boruah SMB, Sahu PP (2021). Quantum image edge detection using improved Sobel mask based on NEQR. Quantum Inf. Process..

[35] Eumelen GJAM, Bosco E, Suiker ASJ, Hermas JJ (2023). Chemo-mechanical model for degradation of oil paintings by amorphous and crystalline metal soaps. Eur. J. Mech.-A/Solids..

[36] Huang J (2022). Personalized college English learning based on deep learning under the background of big data. Comput. Intell. Neurosci..

[37] Chiu MC, Hwang GJ, Hsia LH, Shyu FM (2022). Artificial intelligence-supported art education: A deep learning-based system for promoting university students’ artwork appreciation and painting outcomes. Interact. Learn. Environ..

[38] Qin X (2023). Research on the application of deep learning algorithm based PS design software technology in oil painting teaching. Int. J. Netw. Virtual Organ..

[39] Zhu H (2022). The optimization function of computer image technology in processing oil painting creation. Wirel. Commun. Mobile Comput..

