# Supplementary material for: Oil painting teaching design based on the mobile platform in higher art education
Source: Sci Rep. 2024 Jul 5;14:15531. doi: 10.1038/s41598-024-65103-3 (PMC11226682; doi:10.1038/s41598-024-65103-3)
Supplement: Supplementary file 1 — Supplementary Information. [file 41598_2024_65103_MOESM1_ESM.zip › Code and Description/Code description.docx]

This paragraph describes a Python code for collecting a dataset and downloading 1,000 oil painting images from a specified website. The images are categorized into five different styles: Baroque, Cubism, Impressionism, Renaissance, and Rococo, each containing 200 images. The dataset is split into a 75% training set (150 images per style) and a 25% test set (50 images per style) as per the requirements.
